# Supplementary figures and images for: CALD1 Modulates Gliomas Progression via Facilitating Tumor Angiogenesis
Source: Cancers (Basel). 2021 May 30;13(11):2705. doi: 10.3390/cancers13112705 (PMC8199308; doi:10.3390/cancers13112705)

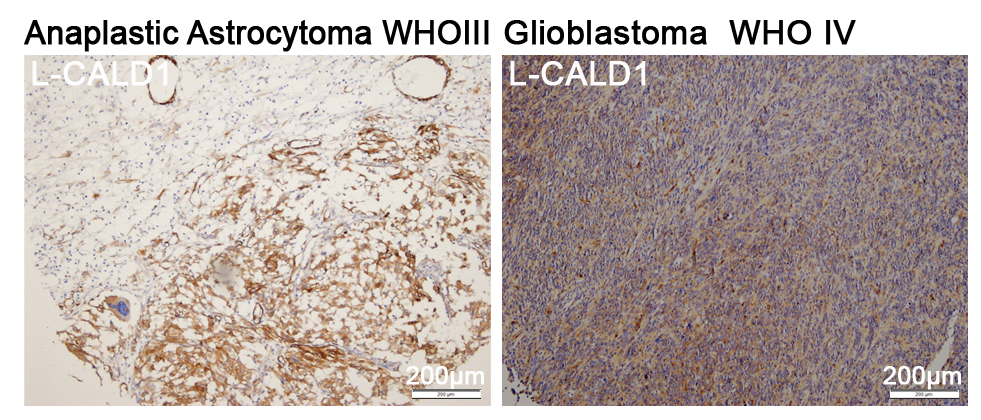

Supplement: Supplementary file 1 [file cancers-13-02705-s001.zip › cancers-1205665_supp.tif]

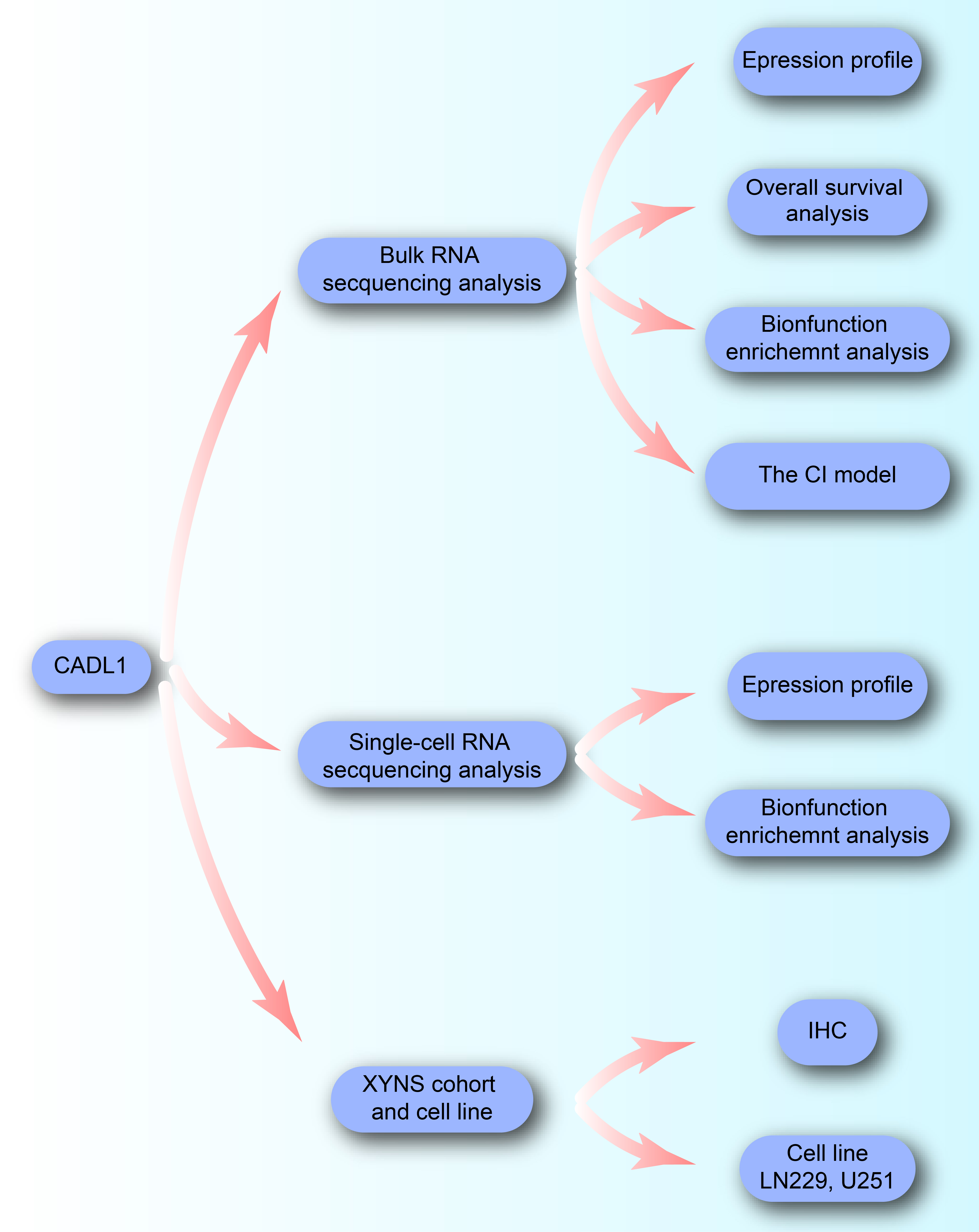

Supplement: Supplementary file 1 [file cancers-13-02705-s001.zip › Sup-fig. 1 Flow chart.jpg]

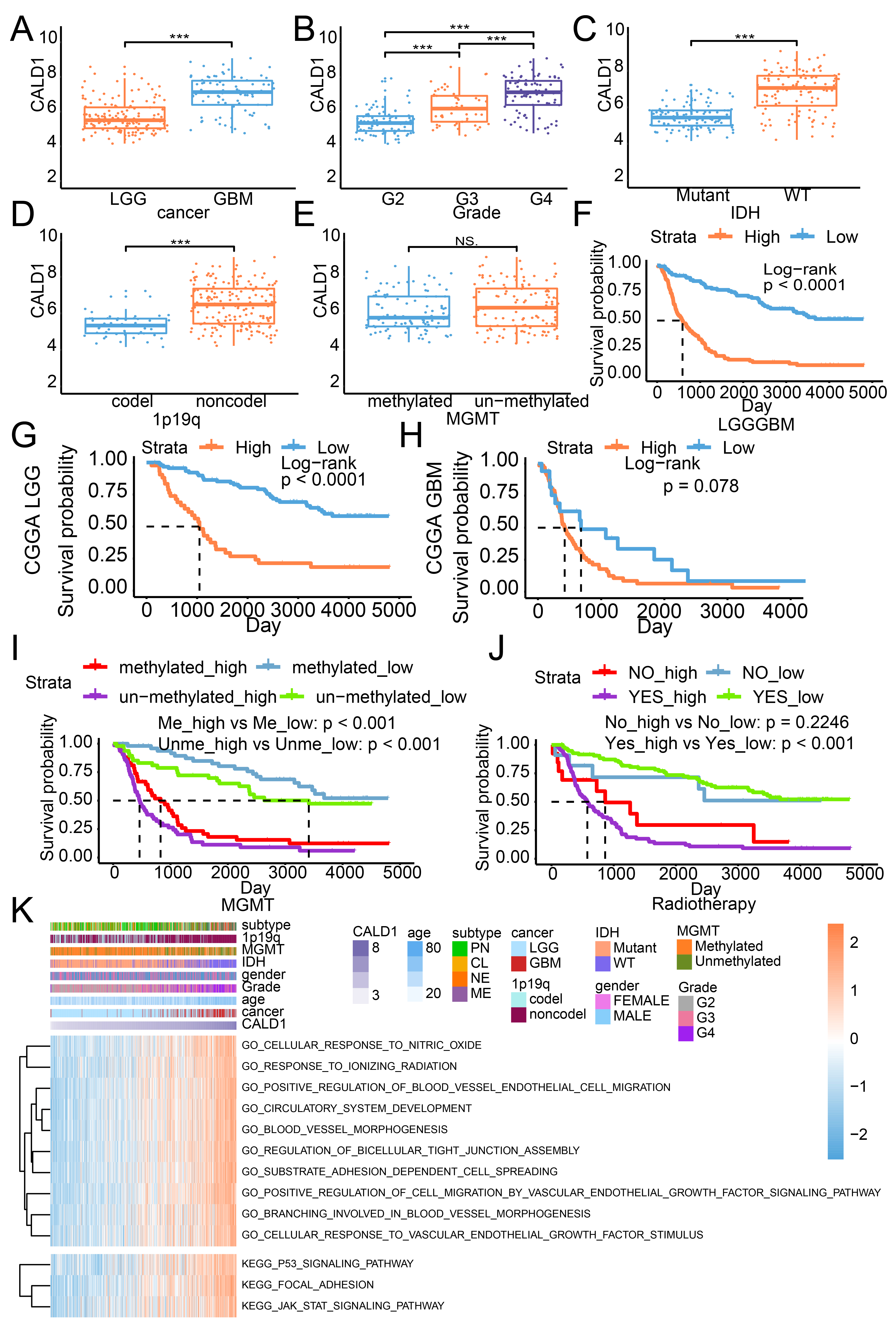

Supplement: Supplementary file 1 [file cancers-13-02705-s001.zip › Sup-fig. 2 CALD1 CGGA.jpg]

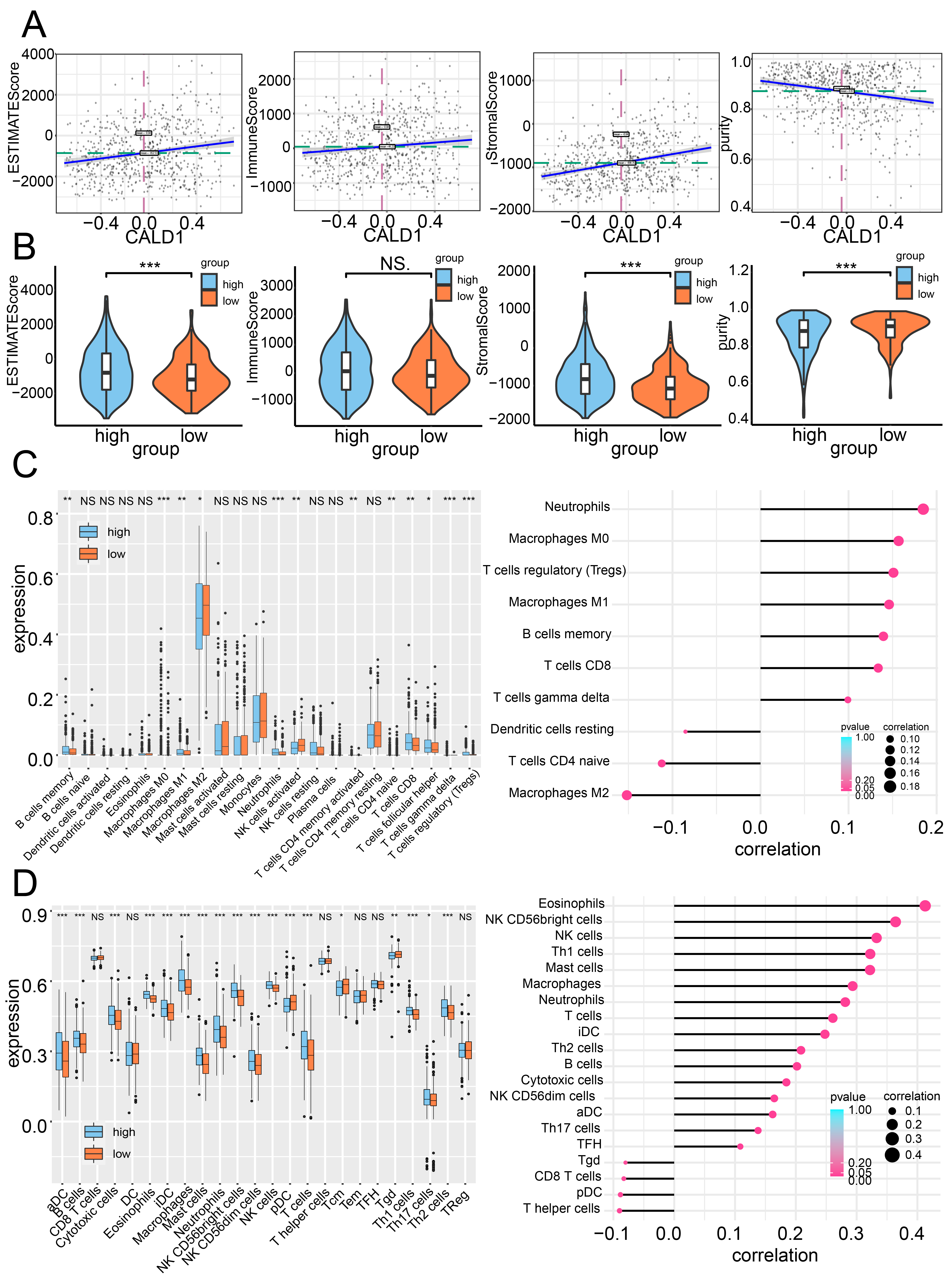

Supplement: Supplementary file 1 [file cancers-13-02705-s001.zip › Sup-fig. 3 immune TCGA.jpg]

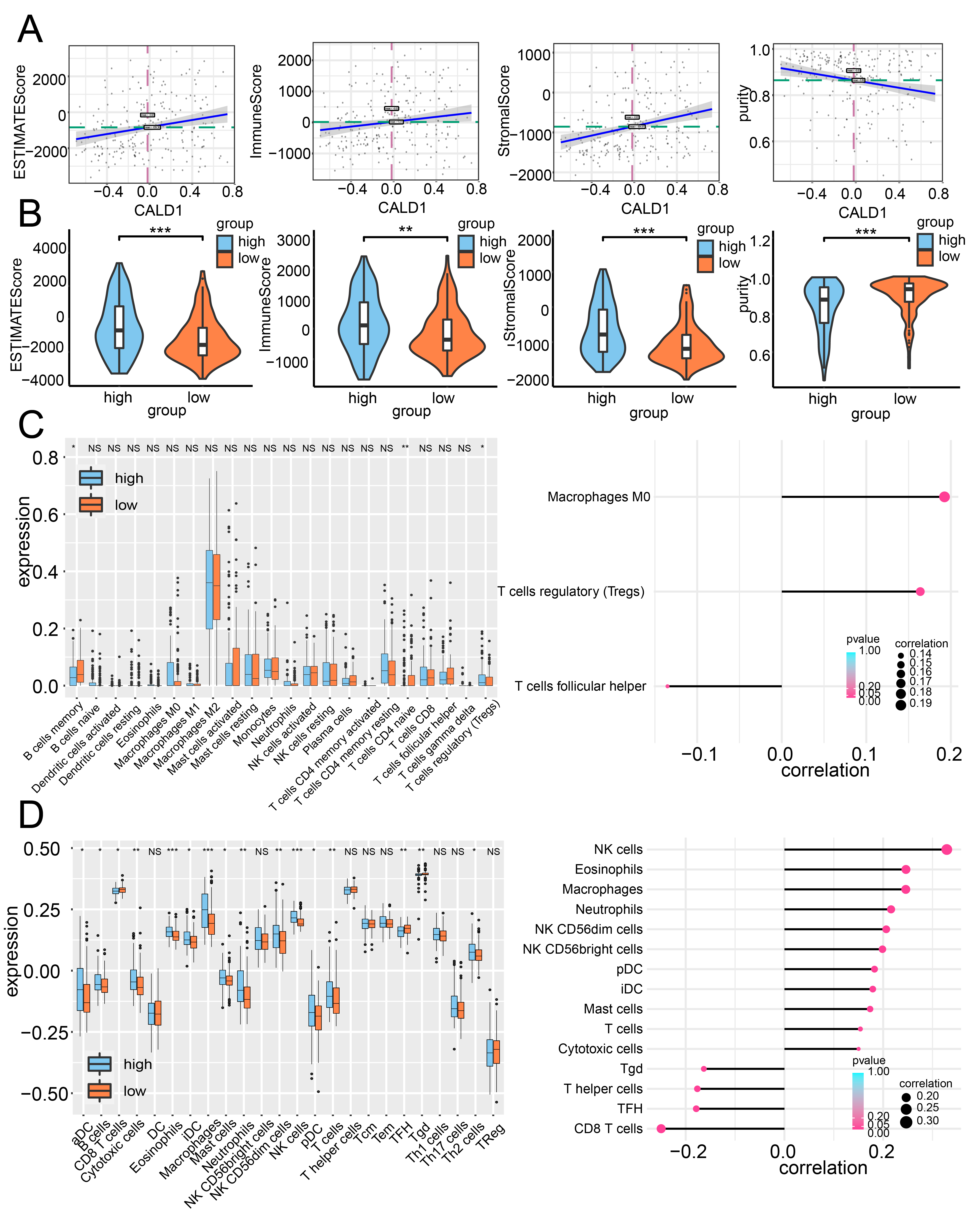

Supplement: Supplementary file 1 [file cancers-13-02705-s001.zip › Sup-fig. 4 immune CGGA.jpg]

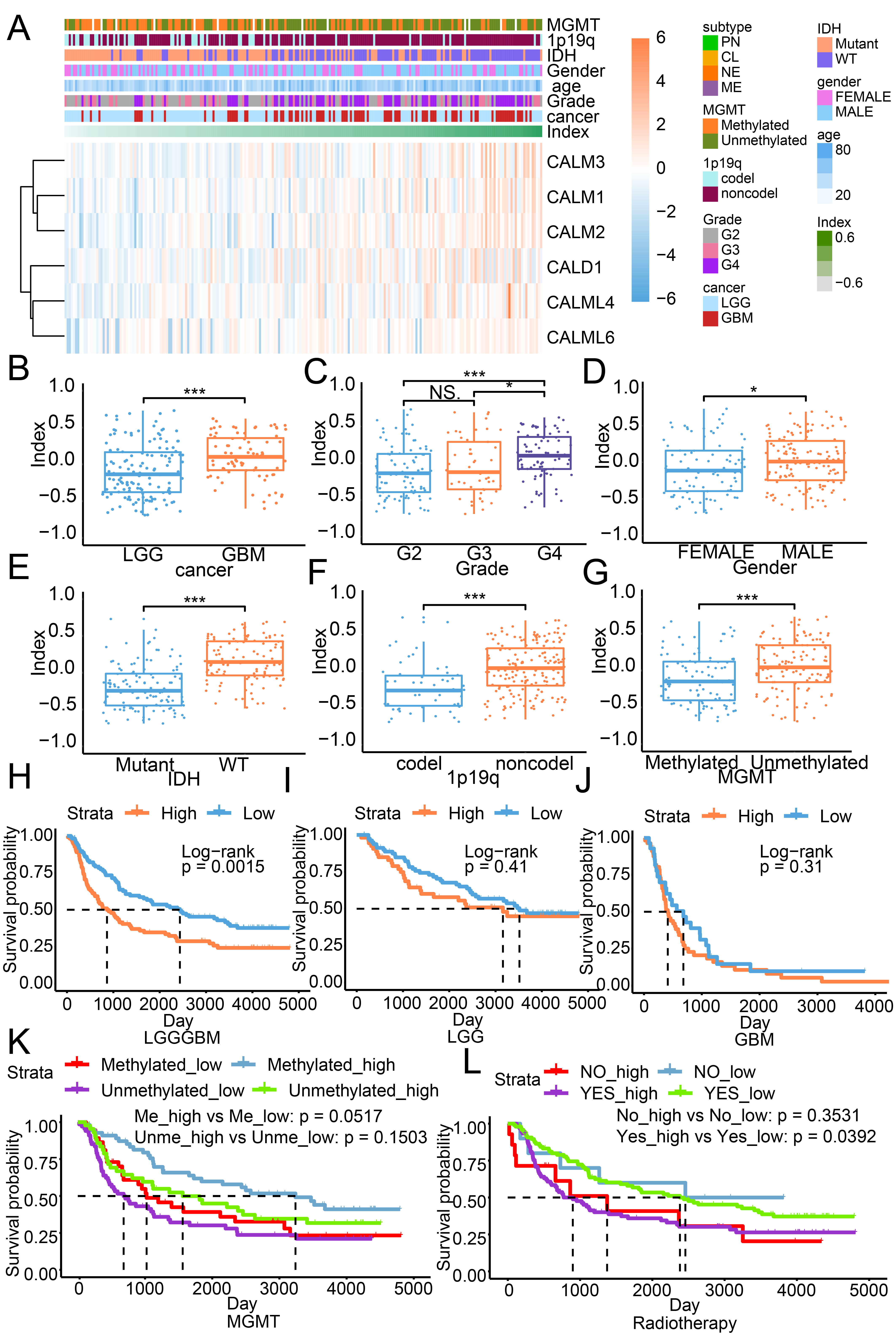

Supplement: Supplementary file 1 [file cancers-13-02705-s001.zip › Sup-fig. 6 Index CGGA.jpg]
